# Letter to the editor RE: “Conservative management of 11 weeks old cervical ectopic pregnancy with transvaginal ultrasound-guided combined methotrexate injection: Case report and literature review”

**DOI:** 10.1016/j.ijscr.2020.03.050

**Published:** 2020-05-08

**Authors:** Antoine Naem, Bashar Al-Kurdy

**Affiliations:** Faculty of Medicine of Damascus University, Damascus, Syria; a Faculty of Medicine of Damascus University, Damascus, Syria; b University Hospital of Obstetrics and Gynecology in Damascus, Syria

Dear Mr. Editor,

The cervical ectopic pregnancy is a very rare condition that accounts for less than 1% of all ectopic pregnancies [1]. Regarding its rarity, the standard management remains controversial. It also depends on the equipment availability and the gynecologist’s experience. The cervical ectopic pregnancy endangers the patient’s life and fertility as well. Even though the cervical ectopic pregnancy is being studied excessively recently, we still need more information to fulfill our knowledge. On this basis, the valuable case reported by Dr. Ozcivit et al. [2] is priceless; especially that it states the outcomes of using local methotrexate injection in treating a relatively advanced cervical ectopic pregnancy. Local methotrexate injection is a uterus-sparing conservative management that helps in avoiding the side effects of systemic methotrexate administration.

In fact, fertility preservation is a major and common demand of most patients suffering from cervical ectopic pregnancy. Recent studies have shown that 75% of these patients desire future fertility [3]. Moreover, the combination between local methotrexate injection and intramuscular methotrexate injection has higher success rate over other management regimens [4]. Other studies have shown that patients treated with local methotrexate injection had short hospital stay (maximum for 24 h), short operating time and uncomplicated recovery [5]. Therefore, the patient’s hospital stay in this case is considered very long and could have been shorter. Patients managed with surgical interventions had longer hospital stay and suffered from sepsis, hemorrhage and other complications [3]. In addition, we strongly believe that Dilatation and Curettage (D&C) should no longer be considered as a therapeutic option in patients suffering from cervical ectopic pregnancy because it was proved that D&C is a potential risk factor of developing a future cervical ectopic pregnancy [6]. In cases of heterotopic pregnancies, sole usage of local methotrexate injection resolved successfully the cervical pregnancy and preserved the intrauterine pregnancy [5]. This may encourage gynecologists to abandon the use of systemic methotrexate administration in the near future. However, the elongated resolution period of serum β-HCG levels, or its peaking at the seventh day post-injection may concern gynecologists, but none of the mentioned events has any clinical significance [5,7]. Finally, unlike what was mentioned by the authors, recent studies have shown that initial serum β-HCG levels fail to predict the management outcomes of any therapeutic method [4]. Nevertheless, initial β-HCG levels might be related to β-HCG resolution period, i.e. higher β-HCG levels at presentation may be accompanied by longer resolution period [5]. In conclusion, initial serum β-HCG levels shouldn’t be considered when choosing the therapeutic intervention. It should be only used as a follow-up method.

## Funding

There were no sources of funding.

## Ethical approval

No ethical approval was needed.

## Consent

No consent was needed.

## Author contribution

Antoine Naem: Reviewed the literature and wrote the manuscript.

Bashar Al-Kurdy: Supervised the writing process and revised the manuscript.

## Registration of research studies

N/A.

## Guarantor

Mr. Antoine Naem.

## Declaration of Competing Interest

All of the authors declared that they have no conflict of interest.

## References

[1] Sepulveda Gonzalez G. (2019). Successful management of heterotopic cervical pregnancy with ultrasonographic-guided laser ablation. J. Minim. Invasive Gynecol..

[2] Ozcivit I.B. (2020). Conservative management of 11 weeks old cervical ectopic pregnancy with transvaginal ultrasound-guided combined methotrexate injection: case report and literature review. Int. J. Surg. Case Rep..

[3] Shah J.S. (2019). Management and reproductive counseling in cervical, caesarean scar and interstitial ectopic pregnancies over 11 years: identifying the need for a modern management algorithm. Hum. Reprod. Open.

[4] Ramkrishna J. (2018). Comparison of management regimens following ultrasound diagnosis of nontubal ectopic pregnancies: a retrospective cohort study. BJOG Int. J. Obstet. Gynaecol..

[5] Gilbert S.B. (2020). Direct methotrexate injection into the gestational sac for nontubal ectopic pregnancy: a review of efficacy and outcomes from a single institution. J. Minim. Invasive Gynecol..

[6] Hoyos L.R. (2019). Risk factors for cervical ectopic pregnancy. J. Gynecol. Obstet. Hum. Reprod..

[7] Koch M. (2018). Management of uterine ectopic pregnancy–local vs. systemic methotrexate. Acta Obstet. Gynecol. Scand..

